# Prevalence and trends in transmitted and acquired antiretroviral drug resistance, Washington, DC, 1999–2014

**DOI:** 10.1186/s13104-017-2764-9

**Published:** 2017-09-11

**Authors:** Annette M. Aldous, Amanda D. Castel, David M. Parenti, the DC Cohort Executive Committee, Alan E. Greenberg, Debra Benator, Princy Kumar, Richard Elion, Maria Elena Ruiz, Angela Wood, Lawrence D’Angelo, Natella Rakhmanina, Sohail Rana, Maya Bryant, Saumil Doshi, Annick Hebou, Ricardo Fernandez, Stephen Abbott, Rachel Hart, Michael Kharfen, Henry Masur

**Affiliations:** 1 Department of Epidemiology and Biostatistics, The George Washington University, Milken Institute School of Public Health, Washington, DC 20037 USA; 2 Division of Infectious Diseases, The George Washington University School of Medicine, 2150 Pennsylvania Avenue, NW, Washington, DC USA

**Keywords:** HIV, Antiretroviral therapy, Drug resistance, Transmitted drug resistance, Acquired drug resistance, Cumulative drug resistance, Prevalence, Washington, DC

## Abstract

**Background:**

Drug resistance limits options for antiretroviral therapy (ART) and results in poorer health outcomes among HIV-infected persons. We sought to characterize resistance patterns and to identify predictors of resistance in Washington, DC.

**Methods:**

We analyzed resistance in the DC Cohort, a longitudinal study of HIV-infected persons in care in Washington, DC. We measured cumulative drug resistance (CDR) among participants with any genotype between 1999 and 2014 (n = 3411), transmitted drug resistance (TDR) in ART-naïve persons (n = 1503), and acquired drug resistance (ADR) in persons with genotypes before and after ART initiation (n = 309). Using logistic regression, we assessed associations between patient characteristics and transmitted resistance to any antiretroviral.

**Results:**

Prevalence of TDR was 20.5%, of ADR 40.5%, and of CDR 45.1% in the respective analysis groups. From 2004 to 2013, TDR prevalence decreased for nucleoside and nucleotide analogue reverse transcriptase inhibitors (15.0 to 5.5%; p = 0.0003) and increased for integrase strand transfer inhibitors (INSTIs) (0.0–1.4%; p = 0.04). In multivariable analysis, TDR was not associated with age, race/ethnicity, HIV risk group, or years from HIV diagnosis.

**Conclusions:**

In this urban cohort of HIV-infected persons, almost half of participants tested had evidence of CDR; and resistance to INSTIs was increasing. If this trend continues, inclusion of the integrase-encoding region in baseline genotype testing should be strongly considered.

## Background

Since 1995, the use of combination antiretroviral therapy (ART) has dramatically improved life expectancy and health outcomes for people infected with HIV, but resistance to antiretroviral drugs (ARVs) undermines their effectiveness [1–4]. Drug resistance may be acquired in response to drug pressure (ADR) or transmitted at the time of infection (TDR). In the United States (US), estimates of TDR prevalence range from 4 to 27% [5–28]. Some reports indicate that resistance to nucleoside and nucleotide analogue reverse transcriptase inhibitors (NRTIs) has remained stable or decreased, while resistance to nonnucleoside reverse transcriptase inhibitors (NNRTIs) and protease inhibitors (PIs) has remained stable or increased [14, 19, 23, 26–28]. Few data are available on the prevalence of resistance to the newer ARV classes: entry/fusion inhibitors (EIs) and integrase strand transfer inhibitors (INSTIs). Two recent studies found no resistance to INSTIs [29, 30]; however, with INSTI-based regimens featuring prominently in the latest US Department of Health and Human Services treatment guidelines [31], increasing resistance to this class is likely.

While TDR has been fairly well documented, fewer data exist on rates of ADR and of cumulative drug resistance (CDR), a term we use to encompass all resistance, whether transmitted, acquired, or of unknown origin. One study of homeless persons in San Francisco found ADR prevalence of 36% [9]. The same study and one other found CDR prevalence rates of 27 and 45%, respectively [9, 32]. These categories of resistance may provide an indication of how well a city is maintaining treatment and adherence in its infected population. Additionally, Tilghman et al. found that high levels of CDR at specific gene locations predicted TDR in the same locations [33].

In Washington, DC, which has an HIV prevalence of 2.5% [34], recent studies of TDR have found that 17–23% of participants had mutations associated with resistance to at least one drug [26, 28] while two earlier studies reported resistance rates of up to 17%, depending on drug class [35, 36]. These findings suggest resistance is common, yet citywide prevalence is unknown. The DC Cohort, a longitudinal observational study of HIV-infected persons receiving outpatient care at 13 clinics throughout Washington, DC [37], affords a unique opportunity to characterize prevalence in a major urban area with a high burden of HIV. With 6743 people enrolled as of December 2014, including 4969 DC residents, the study aims to provide a representative sample of the 16,423 people estimated to be living with HIV in the city [34]. Additionally, the longitudinal nature of the study makes it possible to distinguish, for some participants, between transmitted and acquired drug resistance.

In this analysis, we aimed to describe the prevalence of and trends in ARV drug resistance among DC Cohort participants by category of resistance (TDR, ADR, and CDR); specifically, to measure prevalence of individual drug resistant mutations and to estimate resistance to individual drugs and drug classes. We further sought to examine associations between patient characteristics and the presence of transmitted drug resistance.

## Methods

### Data source and study population

**Fig. 1:**
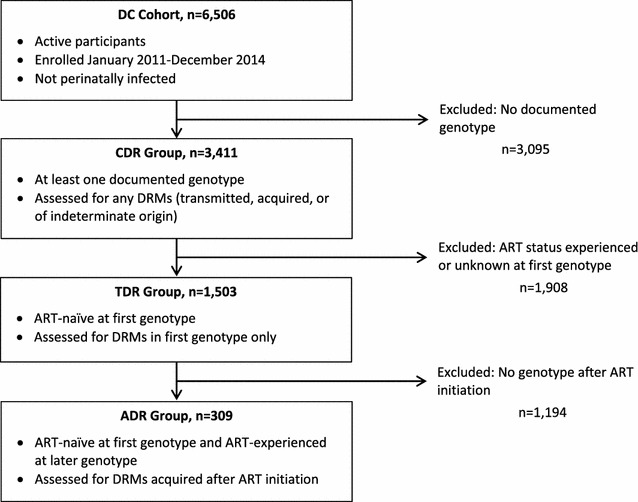
Composition of DC Cohort analysis groups, Washington DC, 1999–2014. CDR, cumulative drug resistance; TDR, transmitted drug resistance; ADR, acquired drug resistance; DRM, drug-resistant mutation; ART, antiretroviral therapy

Enrollment in the DC Cohort began in January 2011. Data on all consenting participants are electronically exported on a monthly basis. Historical data are manually abstracted including genotype and phenotype tests and date of ART initiation where available [38]. For the present analysis, we included all active participants enrolled through December 2014 and not perinatally infected (n = 6506). Our study population included both recently infected individuals and people who had been living with HIV for many years. Participants with any documented genotype test between 1999 and 2014 were included for the estimates of CDR (n = 3411). Those who were documented treatment-naïve at first genotype test were evaluated for TDR (n = 1503). Among the latter group, those who had one or more additional genotype tests after ART initiation were assessed for ADR (n = 309) (Fig. 1). The DC Cohort study was approved by the George Washington University Institutional Review Board (IRB), and all 13 sites received IRB approval to participate in the study.

### Measurement of resistance

Multiple commercial assays were used for the genotype testing, some of which occurred prior to study enrollment. A total of 5993 genotypes were analyzed (LabCorp: 3047; TruGene: 1279; Monogram Biosciences: 621; Quest: 467; other: 579) representing all 13 clinical sites. Although major and minor mutations were available for the reverse transcriptase and protease genes, and sometimes for the envelope and integrase genes, full sequences were not available, and we did not have information on which specific genotypes were evaluated for EI and INSTI resistance. We measured the prevalence of individual drug resistance mutations (DRMs) that were included in the WHO Surveillance Drug Resistance Mutations list [39] or in the 2014 International Antiviral Society-USA (IAS) HIV-1 drug mutations classification [40]. From the latter, we included all bolded amino acid substitutions and all mutations at bolded PI locations. We then interpreted resistance to drugs and drug classes using the IAS classification alone; however, for PIs, since the IAS guidelines identified only major locations and not specific amino acid substitutions, we used the 2014 Stanford HIVDB genotypic resistance interpretation algorithm (Version 7.0), including intermediate and high-level resistance mutations at bolded locations [41]. Phenotypic data were not examined.

To determine the prevalence of TDR, we used the first genotype test for each ART-naïve participant (1503 tests). For ADR, we assessed mutations present in tests after ART initiation (557 tests) and absent in the initial test (309 tests). We did not have complete data on the drug regimen for each participant at the time of the test; therefore specific regimen was not taken into account. The CDR analysis group included all participants in the TDR and ADR groups as well as many more for whom we were not able to ascertain whether mutations were transmitted or acquired. To estimate the prevalence of cumulative drug resistance in this group, we included all DRMs on every test, regardless of treatment status at the time of testing (5993 tests). A participant with a given mutation on any test was considered to have that mutation for the remainder of the study period.

### Analysis

Characteristics of the DC Cohort at enrollment were assessed as frequencies and proportions for categorical variables and as medians and interquartile ranges for continuous variables. To evaluate trends in resistance from 2004 to 2013, we used the Cochran–Armitage test with 2-sided p values. We performed simple and multivariable logistic regression to examine potential associations between patient characteristics and transmitted resistance to any drug class. In the multivariable model, we included, a priori, age at genotype test, race/ethnicity, transmission risk group, and years from HIV diagnosis along with any variables that proved statistically significant with α = 0.05 in bivariate regression. All analysis was conducted using SAS 9.2 (SAS Institute, Cary, North Carolina).

## Results

### Demographics

The median age of DC Cohort participants at enrollment was 48 years. Participants were mostly male (73.8%), non-Hispanic black (76.4%) and infected through male-to-male sex (38.7%) or heterosexual sex (30.7%). Nearly two-thirds had public health insurance (64.9%), and roughly equal numbers of participants received care at hospital-based clinics (48.2%) and community-based clinics (51.8%). Most participants had CD4 counts above 500 cells/μl (51.5%) and viral loads below 400 copies/ml (75.7%) at enrollment; 41.6% of participants had been diagnosed with AIDS. The median interval between HIV diagnosis and consent date was 9.3 years (Table 1). Clinical characteristics at enrollment were not reflected in the resistance results, which were based on genotype tests that were often performed years earlier or later.

**Table 1:** Characteristics of DC Cohort at enrollment, Washington DC, 1999–2014

Characteristic at enrollment^a^	N (%)
All participants	6506 (100)
Age	
<18	17 (0.26)
18–29	693 (10.65)
30–39	1134 (17.43)
40–49	1945 (29.90)
50–59	1948 (29.94)
60+	769 (11.82)
Sex	
Female	1702 (26.16)
Male	4804 (73.84)
Race/ethnicity	
Non-Hispanic black	4972 (76.42)
Non-Hispanic white	904 (13.89)
Hispanic	282 (4.33)
Other	127 (1.95)
Unknown	221 (3.40)
Transmission risk group	
Male-to-male sexual contact (MMS)	2515 (38.66)
Heterosexual contact	1995 (30.66)
Injection drug use (IDU)	458 (7.04)
MMS/IDU	80 (1.23)
Other	121 (1.86)
Unknown/missing	1337 (20.55)
Insurance	
Public	4222 (64.89)
Private	1767 (27.16)
Other	131 (2.01)
Unknown	386 (5.93)
Clinic type	
Hospital-based	3136 (48.20)
Community-based	3370 (51.80)
Clinical status	
HIV	3798 (58.38)
AIDS	2708 (41.62)
CD4 count (cells/µl)	
<50	158 (2.43)
50–199	525 (8.07)
200–349	938 (14.42)
350–499	1285 (19.75)
≥500	3349 (51.48)
Unknown	251 (3.86)
Viral load (copies/ml)	
0–399	4922 (75.65)
400–999	186 (2.86)
1000–9999	366 (5.63)
10,000–49,999	362 (5.56)
50,000–99,999	155 (2.38)
≥100,000	250 (3.84)
Unknown	265 (4.07)
Alcohol use	
Current	915 (14.06)
Previous	924 (14.20)
Never	3038 (46.70)
Unknown	1629 (25.04)
Recreational drug use	
Current	774 (11.90)
Previous	1416 (21.76)
Never	2147 (33.00)
Unknown	2169 (33.34)
Intravenous drug use	
Current	45 (0.69)
Previous	476 (7.32)
Never	3132 (48.14)
Unknown	2853 (43.85)

Characteristic at enrollment^a^	Median (IQR)
HIV diagnosis to consent (years)	9.3 (4.2–16.3)
HIV diagnosis to ART start (years)	1.2 (0.1–6.2)
ART start to consent (years)	3.6 (1.2–7.8)

ART, antiretroviral treatment

^a^Clinical characteristics at enrollment do not correspond to resistance results, which are based on tests performed at other times

### Prevalence

Among the 5993 genotypes analyzed, 5895 were subtype B, 48 were C, 17 were AG, and 33 were other subtypes. In the TDR group (ART-naïve at genotype), prevalence of TDR to any drug class was 20.5%: 7.9% for NRTIs, 11.7% for NNRTIs, 5.7% for PIs, 1.1% for EIs, and 0.9% for INSTIs (Table 2). In the ADR group (genotypes before and after ART initiation), ADR prevalence was 40.5%; while in the CDR group (all participants tested), CDR prevalence was 45.1%. In terms of specific drugs, all three groups were most resistant to efavirenz (TDR: 10.0%; ADR: 24.6%; CDR 27.2%), and nevirapine (TDR: 10.2%; ADR: 23.9%; CDR 27.1%). The ADR and CDR groups also had high levels of resistance to emtricitabine and lamivudine (TDR: 3.1%; ADR: 20.4%; CDR: 24.3%), and abacavir (TDR: 3.5%; ADR: 19.1%; CDR: 24.2%). Among the protease inhibitors, the highest levels of TDR and CDR were to nelfinavir (TDR: 1.9%; ADR: 0.0%; CDR: 7.2%), and of ADR to atazanavir (TDR: 1.8; ADR: 3.2%; CDR: 5.3). No resistance to darunavir was detected as TDR, ADR, or CDR. Resistance to the fusion inhibitor enfuvirtide was found in a few participants (TDR: 1.1%; ADR: 1.0; CDR: 1.5). Maraviroc resistance was not assessed because tropism determination was not available. Mutations conferring resistance to raltegravir (TDR: 0.6%; ADR: 1.6%; CDR: 1.5%) and elvitegravir (TDR: 0.9%; ADR: 0.6%; CDR: 1.3%) were found in all three analysis groups; while in the CDR group only, 4 participants had evidence of resistance to dolutegravir (TDR: 0.0%; ADR: 0.0; CDR: 0.1%). Resistance to three or more classes was 1.2% for TDR, 1.9% for ADR, and 7.1% for CDR.

**Table 2:** Prevalence of resistance to antiretroviral agents, Washington DC, 1999–2014

	Transmitted drug resistance (n = 1503)		Acquired drug resistance (n = 309)		Cumulative drug resistance (n = 3411)	
	n	%	n	%	n	%
NRTIs^a^						
Abacavir	52	3.5	59	19.1	826	24.2
Didanosine	14	0.9	12	3.9	158	4.6
Emtricitabine	47	3.1	63	20.4	828	24.3
Lamivudine	47	3.1	63	20.4	828	24.3
Stavudine	88	5.9	23	7.4	582	17.1
Tenofovir	7	0.5	10	3.2	98	2.9
Zidovudine	82	5.5	17	5.5	524	15.4
NNRTIs^a^						
Efavirenz	151	10.0	76	24.6	929	27.2
Etravirine	19	1.3	11	3.6	212	6.2
Nevirapine	153	10.2	74	23.9	926	27.1
Rilpivirine	42	2.8	24	7.8	331	9.7
PIs^b^						
Atazanavir	27	1.8	10	3.2	181	5.3
Darunavir	0	0.0	0	0.0	0	0.0
Fosamprenavir	18	1.2	5	1.6	118	3.5
Indinavir	17	1.1	2	0.6	175	5.1
Lopinavir	22	1.5	4	1.3	166	4.9
Nelfinavir	29	1.9	0	0.0	247	7.2
Saquinavir	25	1.7	1	0.3	195	5.7
Tipranavir	13	0.9	3	1.0	117	3.4
EIs^a,c^						
Enfuvirtide	17	1.1	3	1.0	52	1.5
INSTIs^a^						
Dolutegravir	0	0.0	0	0.0	4	0.1
Elvitegravir	14	0.9	2	0.6	45	1.3
Raltegravir	9	0.6	5	1.6	50	1.5
Any ARV	308	20.5	125	40.5	1538	45.1
Any NRTI	118	7.9	71	23.0	1013	29.7
Any NNRTI	176	11.7	81	26.2	998	29.3
Any PI	86	5.7	19	6.1	498	14.6
Any EI	17	1.1	3	1.0	52	1.5
Any INSTI	14	0.9	6	1.9	60	1.8
Any 2 classes	67	4.5	42	13.6	582	17.1
Any 3 classes	18	1.2	5	1.6	228	6.7
Any 4 classes	0	0.0	1	0.3	15	0.4

NRTI, nucleoside/nucleotide analogue reverse transcriptase inhibitor; NNRTI, nonnucleoside reverse transcriptase inhibitor; PI, protease inhibitor; EI, entry/fusion inhibitor; INSTI, integrase strand transfer inhibitor

^a^Interpreted using 2014 International Antiviral Society-USA (IAS) HIV-1 drug mutations classification

^b^Interpreted using 2014 IAS classification and 2014 Stanford HIVDB genotypic resistance interpretation algorithm

^c^The 2014 IAS classification did not include maraviroc

**Fig. 2:**
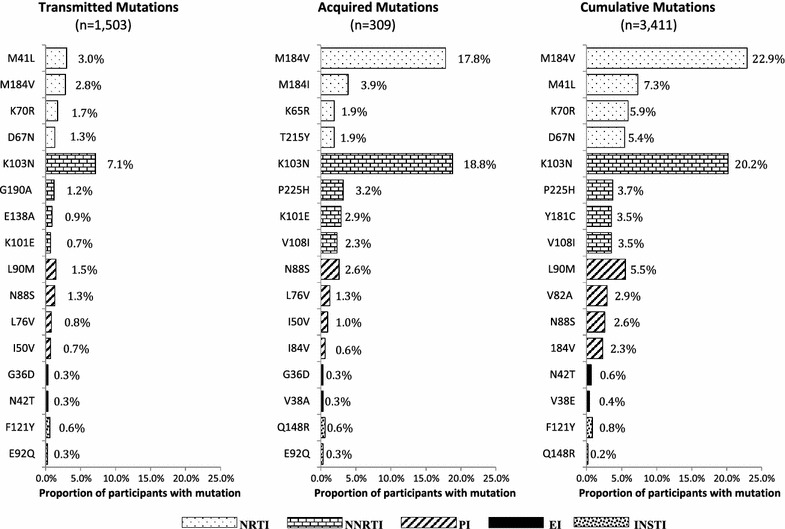
Most prevalent drug-resistant mutations for each analysis group by drug class, Washington DC, 1999–2014. NRTI, nucleoside/nucleotide analogue reverse transcriptase inhibitor (*top 4*); NNRTI, nonnucleoside reverse transcriptase inhibitor (*top 4*); PI, protease inhibitor (*top 4*); *EI*, entry/fusion inhibitor (*top 2*); INSTI, integrase strand transfer inhibitor (*top 2*)

The prevalence of the K103N mutation, associated with resistance to NNRTIs, was high for all three analysis groups (TDR: 7.1%; ADR: 18.8%; CDR: 20.2%) (Fig. 2). Prevalence was also high for NRTI-associated mutations M41L (TDR: 3.0%; ADR: 1.0%; CDR: 7.3%) and M184V (TDR: 2.8%; ADR: 17.8%; CDR: 22.9%). Among protease-associated mutations, L90M (TDR: 1.5%; ADR: 0.0%; CDR: 5.5%) was most prevalent in the TDR and CDR groups, and N88S (TDR: 1.3%; ADR: 2.6%; CDR: 2.6%) in the ADR group. Integrase-associated mutations were detected at nine sites: primarily at F121Y (TDR: 0.6%; ADR: 0.3%; CDR: 0.8%), E92Q (TDR: 0.3%; ADR: 0.3%; CDR: 0.2%), Q148R (TDR: 0.0%; ADR: 0.6%; CDR: 0.2%), and N155H (TDR: 0.0%; ADR: 0.0%; CDR: 0.2%). On the *env* gene, the most common mutation was N42T (TDR: 0.3%; ADR: 0.0%; CDR: 0.6%).

### Time trends

**Fig. 3:**
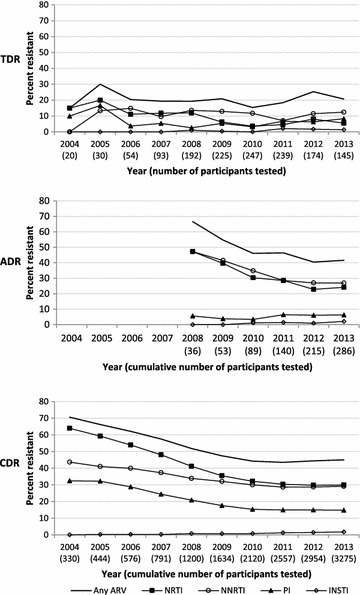
Trends in antiretroviral resistance by drug class, Washington DC, 2004–2013. TDR, transmitted drug resistance; ADR, acquired drug resistance; CDR, cumulative drug resistance. Results for 2014 are not shown due to incomplete data at the time of analysis

From 2004 to 2013, TDR was fairly stable around 20% (15.0–20.7%; p = 0.76), with a marked decrease for NRTIs (15.0 to 5.5%; p = 0.0003) and a small increase for INSTIs (0.0–1.4%; p = 0.04) (Fig. 3). Over the same time period, the proportion of newly diagnosed participants who had a genotype test within the first year of diagnosis steadily increased [5.5–65.9%; p < 0.0001 (data not shown)]. For ADR, the number of participants tested prior to 2008 was too small to permit meaningful analysis (fewer than 10 per year). The prevalence of ADR decreased from 66.7% in 2008 to 41.6% in 2013 (p = 0.003). ADR also decreased significantly for NRTIs (47.2 to 24.1%; p = 0.0004) and NNRTIs (47.2 to 26.9%; p = 0.002). Resistance to PIs rose slightly (5.6–6.3%; p = 0.25), but the difference was not significant and as noted above, no resistance was found to darunavir, which has perhaps the highest barrier to resistance. CDR prevalence to any drug class declined significantly from 70.6% in 2004 to 45.0% in 2013 (p < 0.0001). The trend was also significant for NRTIs (63.9 to 29.9%; p < 0.0001), NNRTIs (43.6 to 29.1%; p < 0.0001), PIs (32.4 to 14.8%; p < 0.0001), and to any 2 (33.6 to 17.0%; p < 0.0001) or 3 (17.9 to 6.9%; p < 0.0001) drug classes, while resistance increased for EIs (0.0–1.5%; p < 0.0001), INSTIs (0.0–1.8%; p < 0.0001), and any four drug classes (0.0–0.4%; p < 0.0001).

### Logistic regression analysis

We decided a priori to include age at test, race/ethnicity, transmission risk group and years from HIV diagnosis in the multivariable regression model. Based on a statistically significant association at the α = 0.05 level in bivariate regression analysis, we added clinic type to the model. In multivariable analysis, TDR was not predicted by time between HIV diagnosis and genotype, age at genotype or race/ethnicity. Although not statistically significant, individuals infected through injection drug use (OR 1.53; 95% CI 0.79–2.97) and those receiving HIV care at community-based clinics (OR 1.27; 95% CI 0.95–1.72) were more likely to have transmitted resistance than individuals infected through male-to-male sexual contact and participants cared for at hospital-based clinics, respectively.

## Discussion

To our knowledge, we are the first to report positive findings of mutations associated with transmitted resistance to INSTIs (0.9%) and EIs (1.1%). We also found evidence of resistance to these classes among participants analyzed for ADR and CDR as well as significantly increasing trends for cumulative resistance to both classes and transmitted resistance to INSTIs. Unfortunately we were not able to determine which genotypes included the INSTI and EI encoding regions, and so we report prevalence among all genotypes assessed; thus, our rates are underestimates of prevalence for these classes. The emergence of resistance to INSTIs is likely due to their increasingly widespread use in clinical practice as well as their earlier use in clinical trials. Several DC sites participated in registration trials for all three INSTIs, the first of which (raltegravir) was FDA-approved in 2007; and by the end of 2014, approximately 10% of DC Cohort participants were on INSTI-based regimens. Surprisingly, resistance to elvitegravir—as interpreted using the IAS guidelines—was higher than to raltegravir, although the latter was introduced earlier. This was mainly attributable to the presence of E92Q and T66I mutations. In the most recent US Department of Health and Human Services (DHHS) treatment guidelines, four of the five recommended regimens for ART-naïve patients were INSTI-based, while inclusion of the integrase region in routine genotype testing was still optional [31]. Given the emergence of INSTI resistance in the Washington, DC area, baseline resistance testing for integrase inhibitors should be strongly considered.

The TDR prevalence of 20.5% found in this analysis was comparable to rates reported throughout the US and in Washington, DC. We were surprised to find that 18 participants analyzed for TDR had mutations associated with resistance to three drug classes. Review of the medical records for these 18 participants is warranted to confirm that the recorded dates are accurate. Our findings of no significant association between TDR and sex, race/ethnicity, or transmission risk group support those of most [11, 14, 21, 28], but not all [10] previous studies. However, resistance appeared to be higher among injection drug users (IDUs), and although the difference was of borderline significance, it is plausible given that IDUs may have more barriers to adherence and retention in care [42].

Cumulative drug resistance may serve as a measure of a community’s burden of ARV resistance and, like community viral load, may reflect the success of treatment and adherence in that community [43]; however, these results should be interpreted with caution. First, the decrease in CDR observed between 2006 and 2010 was probably due in part to the dilution effect of increased resistance testing among newly diagnosed individuals following the 2007 DHHS recommendations [31]. Second, because patients on treatment generally have genotype testing performed when treatment fails, the prevalence of resistance in tested individuals may be higher than in the overall population of persons infected with HIV. To avoid this overestimation of resistance, others have included all treatment-experienced patients in the denominator [44] or used modeling to extend resistance estimates from tested to untested individuals [45]. By assuming that untested individuals do not have resistance, the former approach underestimates prevalence. Using this method, we found that resistance appeared to increase as the proportion of participants tested increased dramatically over the time period (results not shown). That is, the degree of underestimation decreased over time, and the resulting apparent increase in resistance was misleading. In future analysis, we hope to model resistance in the overall Cohort taking into account the rate of testing and other secular trends. Third, our measurements were based on genotype tests that do not detect minority or archived HIV strains and thus, may underestimate the true prevalence of resistance. Furthermore, some transmitted archived strains may not have been detected until after treatment was initiated, resulting in misclassification of TDR as ADR. However, since our estimates for ADR and CDR were cumulative, we maximized our ability to include archived strains within the limitations of the tests.

Other strengths of this study include the large size and representative, citywide composition of the DC Cohort, together with the availability of genotypic, demographic, and clinical data. The long-term use of INSTIs in the study population provided early evidence of resistance to this drug class, while the longitudinal data allowed us to assess acquired and cumulative resistance in a large cohort.

## Conclusions

In this urban cohort of HIV-infected persons in care, almost half of participants tested had evidence of CDR, and resistance to INSTIs was increasing. If this trend continues, inclusion of the integrase-encoding region in routine genotype testing may become advisable. With new treatment guidelines recommending immediate initiation of ART for most people, innovations to promote adherence, such as co-formulations and longer-acting regimens, will be more critical than ever. Continued close surveillance of transmitted and acquired resistance will measure the success of these efforts and inform future testing and treatment guidelines.

## Abbreviations

ADR: acquired drug resistance
ART: antiretroviral therapy
ARV: antiretroviral
CDR: cumulative drug resistance
DRM: drug resistance mutation
EI: entry/fusion inhibitor
IAS: International Antiviral Society
IDU: injection drug user
INSTI: integrase strand transfer inhibitor
IRB: institutional review board
MMS: male-to-male sex
NNRTI: nonnucleoside reverse transcriptase inhibitor
NRTI: nucleoside/nucleotide analogue reverse transcriptase inhibitor
PI: protease inhibitor
TDR: transmitted drug resistance
